# The Prognostic Significance of Anisomycin-Activated Phospho-c-Jun NH2-Terminal Kinase (p-JNK) in Predicting Breast Cancer Patients’ Survival Time

**DOI:** 10.3389/fcell.2021.656693

**Published:** 2021-03-09

**Authors:** Li Chen, Xuantong Zhou, Xiangyi Kong, Zhaohui Su, Xiangyu Wang, Sen Li, Aiping Luo, Zhihua Liu, Yi Fang, Jing Wang

**Affiliations:** ^1^Department of Breast Surgical Oncology, National Cancer Center/National Clinical Research Center for Cancer/Cancer Hospital, Chinese Academy of Medical Sciences and Peking Union Medical College, Beijing, China; ^2^State Key Lab of Molecular Oncology, National Cancer Center/National Clinical Research Center for Cancer/Cancer Hospital, Chinese Academy of Medical Sciences and Peking Union Medical College, Beijing, China; ^3^Center on Smart and Connected Health Technologies, Mays Cancer Center, School of Nursing, UT Health San Antonio, San Antonio, TX, United States; ^4^Department of General Surgery, The Affiliated Cancer Hospital of Zhengzhou University, Zhengzhou, China

**Keywords:** p-JNK, anisomycin, p-STAT3, neoadjuvant chemotherapy, breast cancer

## Abstract

This study aims to investigate the prognostic significance of p-JNK in breast cancer patients receiving neoadjuvant chemotherapy (NACT) and analyze the relationship between anisomycin, p-JNK. A total of 104 breast cancer patients had NACT were enrolled in this study. The western blot and immunohistochemistry assays were used to determine the protein expressions of p-JNK in human breast cancer cell lines and patients’ cancer tissues. The chi-square test and Fisher’s exact test were adopted to gauge the associations between breast cancer and clinicopathological variables by p-JNK expression, whereas the univariate and multivariate Cox proportional hazards regression models were used to analyze the prognostic value of p-JNK expression. The Kaplan-Meier plots and the log-rank test were adopted to determine patients’ disease-free survival (DFS) and overall survival (OS). Findings indicated that the p-JNK expression had prognostic significance in univariate and multivariate Cox regression survival analyses. Results of log-rank methods showed that: (1) the mean DFS and OS times in patients with high p-JNK expression were significantly longer than those in patients with low p-JNK expression (χ^2^ = 5.908, *P* = 0.015 and χ^2^ = 6.593, *P* = 0.010, respectively). p-JNK expression is a significant prognostic factor that can effectively predict the survival in breast cancer patients receiving NACT. Treatment with the JNK agonist anisomycin can induce apoptosis, lead to increased p-JNK expression and decreased p-STAT3 expression. Moreover, the p-JNK expression was inversely correlated with p-STAT3 expression.

## Introduction

Breast cancer is the most common malignancy for females all over the world (Santucci et al., 2020; Siegel et al., 2020). Accounting for approximately 30% of all new cancer cases, breast cancer is the leading cause of cancer-related morbidity and mortality worldwide among women from 20 to 59 years old (Santucci et al., 2020; Siegel et al., 2020). Situations in China are even worse. It is estimated that around 9.6% breast cancer deaths occurred in China (Fan et al., 2014; Dibden et al., 2020). Since the 1990s, the incidence of breast cancer in China has been doubling the global rates (11.6%) (Linos et al., 2008; Bray et al., 2018). Researchers further estimate that, by 2021, for women aged 55–69 years old, breast cancer case numbers in China are expected to jump from less than 60 cases to more than 100 cases per 100,000, resulting in an unprecedented total of 2,500,000 cases (Linos et al., 2008; Bray et al., 2018). These daunting numbers, undoubtedly, call for timely research and innovations that can effectively address potential needs and wants of Chinese breast cancer patients.

With the help of screening tests, such as mammography and magnetic resonance imaging, an increasing number of breast cancer patients have benefited from an early stage diagnosis. These diagnoses, in turn, can often be effectively addressed by advanced diagnosis treatments (e.g., fine needle aspiration and core needle biopsy). However, it is important to note that approximately 20–25% of patients are diagnosed with advanced breast cancer (e.g., metastatic breast cancer) that requires more sophisticated cancer care and management (Harbeck and Gnant, 2017). In the last several decades, surgery, often combined with adjuvant chemoradiotherapy, has been used to treat patients with advanced breast cancer, distant metastasis, or local recurrence (Harbeck et al., 2019). However, to date, no treatment is available that has the potential to cure advanced breast cancer, or significantly prolongs long-term patient survival. This, in turn, calls for more effective treatment strategies that can improve breast cancer patients’ quality of life and prolong their survival time.

Neoadjuvant chemotherapy (NACT) is regarded as one of most effective before-operation therapies in treating cancer, ranging from esophageal carcinoma, breast cancer, to colorectal cancer (Das, 2017; Derks and van de Velde, 2018; Montemurro et al., 2020). For early breast cancer, NACT can help patients avoid mastectomy by shrinking tumor volume (Mieog et al., 2007). For patients who need mastectomy, NACT can help increase breast-conserving surgery’s success rates as well as the likelihood of eradicating micro metastatic disease (Asselain et al., 2018; Caldana et al., 2018). Moreover, NACT can provide useful information about local tumor’s chemosensitivity to different chemotherapy regimens *in vivo*, helping clinicians and oncologists to make evidence-based decisions of subsequent drug selection (Adamson et al., 2019; Pathak et al., 2019).

Although a plethora of NACT regimens have been applied in the treatment of breast cancer, there has yet to be an internationally recognized NACT regimen for treating advanced breast carcinoma (Klein et al., 2019; Shien and Iwata, 2020). Some histologic and immunologic indicators, such as hormone receptor (HR), human epidermal growth factor receptor-2 (HER-2), and Ki-67 of breast cancer, have significant implications in the prognosis and choice of treatment for breast cancer (Ge et al., 2015). However, some cases received NACT failed to achieve tumor regression, furthermore, the prognosis of NACT-refractory patients would become worse due to the delay in the curative treatment (Kunnuru et al., 2020). Hence, there is an urgent need to identify novel and sensitive indicators, such as mitogen-activated protein kinase (MAPK), to improve therapeutic options for breast cancer patients and provide better treatment measures.

A growing body of research indicates that MAPK plays an important role in regulating inflammatory responses, cell proliferation and differentiation, stress responses, apoptosis, and immune response (Chen and Ávila, 2019; Wu et al., 2020). MAPK is often discussed in light of its major subfamilies, which include the extracellular signal-regulated kinases (ERKs), c-Jun NH2-terminal kinases (JNKs), and p38 MAPK isoforms as well as its activated expressions (p-ERK, p-JNK, and p-p38) (Davidson et al., 2006). Activation of MAPK is often followed by a cascade of sequential phosphorylation events, such as the phosphorylation of MAPKs on threonine and tyrosine residues by specific upstream MAPK kinases (MEKs or MKKs) (Oh et al., 2020).

Among MARK subfamilies, ERKs are largely activated by growth factor signals, while JNKs and p38 are largely activated by a spectrum of stress related stimuli (Haque et al., 2019; Farkhondeh et al., 2020). It is universally acknowledged that ERKs promote cell growth, proliferation, differentiation, while JNKs and p38 mediate apoptotic signals. The JNKs are the major protein kinases that regulate a variety of physiological processes, including cell proliferation, differentiation, and survival, inflammatory responses, as well as morphogenesis. Subsequently, the JNK pathway is associated with a number of disease states, including inflammatory, diabetes, neurodegenerative disorders, and cancer. It is becoming increasingly clear that the persistent activation of JNKs is closely related to cancer development, progression, and metastasis (Izadi et al., 2020; Xu and Hu, 2020). This realization, in turn, has made JNKs attractive as potential drug targets for therapeutic interventions with small molecule kinase inhibitors, such as ATP-competitive and ATP-non-competitive JNK inhibitors (Oh et al., 2019; Li et al., 2020).

Some studies have indicated that anisomycin is a potent protein synthesis inhibitor, and have the potential to interfere with protein and DNA synthesis by inhibiting peptidyl transferase or the 80S ribosome system (Li et al., 2012). Anisomycin is a key JNK activator that is known for its ability to increase levels of phospho-JNK (p-JNK) (Li et al., 2012). Anisomycin was widely used as an agonist for p38 mitogen-activated protein kinase (p38 MAPK) and Jun-NH2 terminal kinase (JNK), as it can induce apoptosis through the activation of the p38 MAPK and JNK signaling pathways (Nie et al., 2020). Several reports suggest that JNK can be used to study the formation of primary tumors as a tumor suppressor, where JNK serves an inhibitory role by mediating the activation of apoptosis (Reno et al., 2009).

The activated JNK phosphorylates various substrates, such as p53, nuclear factor-activated T cells (NFAT) and signal transducer and activator of transcription 3 (STAT3), could result in the stimulation of a series of apoptotic signaling cascades (Cellurale et al., 2012). JNK can also activate the phosphorylation of the STAT-3 at tyr-705 (Yacobi and Levy, 1975). However, activation of c-Jun NH_2_-terminal kinase (JNK) and p-JNK has not been studied in breast cancer undergoing NACT, and the expression and clinical role of p-JNK in breast cancer are unknown at present. Hence, we aim to use anisomycin, a potent activator of JNK, to (1) examine the role played by JNK in breast cancer cells and (2) analyze the expression of p-JNK in breast malignant transformation and in breast cancer patients with clinical follow-up.

## Materials and Methods

### Patients and Samples

In this study, 104 archived formalin fixed paraffin embedded (FFPE) breast cancer samples and 65 FFPE adjacent normal breast tissues were obtained from the Cancer Hospital Chinese Academy of Medical Sciences in China. All enrolled cases were diagnosed with the histology by core needle biopsy, and had received NACT between June of 2009 to December of 2015. Treatment details (i.e., clinical and demographic data) for all patients were extracted from the patients’ medical records. Patients’ clinical and pathological stages were defined in accordance with the eighth edition of the American Joint Committee Cancer Staging Manual (AJCC) (Abdel-Rahman, 2018).

#### Ethical Approval and Informed Consent

This study was approved by the ethics committee of the Cancer Hospital Chinese Academy of Medical Sciences and was performed within the standards of the Declaration of Helsinki as well as its later amendments for medical research involving human subjects. Written informed consent was obtained from all patients before the study.

### Classification Standard and Response Evaluation

The eighth edition of the AJCC and the Union for International Cancer Control (UICC) were used to evaluate the Tumor Node Metastasis (TNM) stage groupings (Abdel-Rahman, 2018; Kandori et al., 2019). Molecular subtypes of breast cancer were divided into Luminal A, Luminal B HER2-positive, Luminal B HER2-negative, HER2-enriched and Triple negative (Zhao et al., 2020). The Miller and Payne grade (MPG) was used to estimate the histological response, and categorized into five grades that are in line with the number of tumor cells in excision/mastectomy specimens compared with the pretreatment core biopsy. Histologic tumor grades were accessed by the Elston-Ellis modification of Scarff-Bloom-Richardson grading system, and based on three factors (Phukan et al., 2015): (1) gland formation, (2) nuclear features, and (3) mitotic activity. The response rate was defined based on the Response Evaluation Criteria in Solid Tumors (RECIST) guidelines, and were stratified into four groups: (1) complete response (CR) was defined as the complete remission of the tumor; (2) partial response (PR) was defined as least a 50% decrease in the tumor volume; (3) stable disease (SD) was defined as a steady state or a response less than 50%; and (4) progression of disease (PD) was defined as an unequivocal increase of at least 25% in the tumor volume (Freites-Martinez et al., 2020). The sum of CR and PR forms the clinical objective response rate, the sum of SD and PD defines the non-clinical response rate, whereas and the sum of CR, PR and SD constitutes the clinical benefit rate.

### Reagents and Materials

Anisomycin was purchased from TargetMol (Shanghai, China). Dulbecco’s modified Eagle’s medium (DMEM) and fetal bovine serum (FBS) for cell culture were purchased from Gibco-BRL (Grand Island, NY, United States). DMEM: F12 (1:1 Mix) and horse serum for cell culture were purchased from Beijing fine workmanship industry Biotechnology Co., Ltd (Beijing, China) and Gibco BRL (Grand Island, NY, United States), respectively. Cell Counting Kit-8 (CCK-8) reagents were purchased from Dojindo (Tokyo, Japan). The anti-PARP antibody (9542), anti-caspase3 antibody (9662), anti-cleaved caspase-3 antibody (9664), anti-STAT3 antibody (9132) and anti-p-STAT3 antibody (9131) were purchased from Cell Signaling Technology (CST, Danvers, MA, United States). The anti-p-JNK1/2/3 antibody (AP0631) was purchased from ABclonal Technology (Wuhan, China). The anti-β-actin antibody (A5316) were purchased from Sigma (St. Louis, MO, United States).

### Cell Culture

Human breast cancer cell lines (MDA-MB-231, MDA-MB-436, BT549, Hs578T) were obtained from the Beijing Institute of Genomics in the Chinese Academy of Sciences (Beijing, China). MDA-MB-231 cells were cultured in Leibovitz’s L-15 medium supplemented with 10% fetal bovine serum and L-glutamine. MDA-MB-231 cells were maintained at 37°C in a humidified cell incubator without CO_2_. BT549 was cultured in RPMI 1640 medium supplemented with 10% fetal bovine serum, penicillin (100 U/ml) and streptomycin (100 μg/ml). MDA-MB-436 and Hs578T cells were conventionally preserved in DMEM, which contained 10% FBS, 100 U/mL penicillin, 100 μg/mL streptomycin, 4.5 g/L D-Glucose, 5 ml L-Glutamine, and 110mg/L Sodium Pyruvate.

Human mammary epithelial cell line (184B5) was obtained from the Beijing Institute of Genomics in the Chinese Academy of Sciences (Beijing, China). And this cell line conventionally preserved in DMEM: F12 (1:1 Mix), which contained 5% horse serum, with 15 mM HEPES, 2mM L-glutamine, 100 U/mL penicillin and 100 μg/mL streptomycin, 20 ng/ml EGF, 100 ng/ml cholera toxin, 0.01 mg/ml insulin, 500 ng/ml hydrocortisone. Most of the cells were stored in a humidified cell incubator (at 37°C with 5% CO_2__)_, except MDA-MB-231.

### IC50 of Anisomycin in Human Breast Cancer Cell Lines

In our study, in comparison with the controls, drug concentration required to inhibit exactly 50% of the cell viability is considered as the median inhibitory concentration (IC50). The relative cell viability (%) was calculated using the equation ODT/ODC × 100% (where ODT represents the absorbance of the treatment group, and ODC represents the absorbance of the control group). IC50 values were estimated from the concentration-response curve. Briefly, the appropriate number of cells was plated in each well of a 96-well plate and exposed to different concentrations of anisomycin for 48 h. Subsequently, the Cell Counting Kit-8 (CCK-8) reagents were added, and the cells were incubated for 1 h at a dilution of 1:10. At the end of the incubation, using a microplate reader (BioTek, Winooski, VT, United States), the absorbance in each well was measured at 450 nm.

### Apoptosis and Western Blot

Apoptosis was analyzed with the FITC Annexin V Apoptosis Detection Kit (BD biosciences, CA, United States). Western blot was performed according to the standard protocol. β-actin was used as an endogenous control. The antibodies used for western blot in this study were listed with dilutions: PARP, caspase3, cleaved caspase3, p-JNK STAT3, and STAT3 (1:1000) as well as β-actin (1:5000).

### Immunohistochemistry (IHC)

Breast tumors and normal samples used for immunohistochemical analyses were collected from breast cancer patients treated at the Cancer Hospital Chinese Academy of Medical Sciences between June of 2009 to December of 2015. Solutions made of 10% formaldehyde were used to treat fresh tissue specimens; formalin-fixed (pH 7.0) and paraffin-embedded archival tumor tissue of each patient were adopted in this study. 5-μm sections were cut from paraffin-embedded blocks for H&E staining and immunohistochemistry. Then they were dewaxed in xylene and dehydrated in an alcohol gradient of 100, 95, 85, and 70%. To deparaffinize, the slides were washed three times for a duration of 5 min. The endogenous peroxidase activity was blocked by incubating it in 0.3% hydrogen peroxide following methanol for 30 min at 37°C. To provide stable pH, we used phosphate buffered saline (PBS) to wash the slides three times for 5 min. Antigen retrieval was achieved by soaking the slices into citrate buffer at 95°C for 15 min, and then blocked with 10% goat serum albumin and incubated with primary antibodies overnight in a chamber with desired levels of moisture. After washing by PBS, the slides were incubated with a secondary antibody at room temperature for an hour before it was washed by PBS once again. Diaminobenzidine (DAB) was used as a chromogen, and the sections were counterstained with hematoxylin. The arrays were scanned by the Aperio Image Scope system (Leica Biosystems, United States), The antibody used for immunohistochemistry assay (IHC) in this study were listed with dilution: p-JNK (1:100). The immunoreactivity of the p-JNK protein were scored on the basis of the intensity of the predominant cytoplasmic staining area using the following classification system: 0, negative; 1, weakly-positive; 2, median-positive; 3, strongly-positive. All specimens were evaluated by two investigators blinded to the clinical information of the patients.

### Follow-Up

All patients included in this study had postoperative follow-ups in the hospital inpatients or outpatients every 3 months for the first to second year, every 6 months for the third to fifth year after surgery, and then every 12 months from fifth year forward. Disease-free survival (DFS) was calculated from the date of the surgery operation to the time when either local recurrence, distant metastases, relapse, or death (from any reason) occurred. Overall survival (OS) was defined as the time from the date of operation to the date of death from any reason or final follow-up. Survival duration was measured from the date of the operation to death or at the final follow-up.

### Statistical Analysis

Statistical analysis was performed using GraphPad Prism 8.0 and SPSS software version 17.0 (SPSS, Inc., Chicago, IL, United States). The clinicopathologic categorical variables were performed as frequencies and percentages (%). Based on the context, either chi-square test or Fisher’s exact test was adopted to evaluate the associations between clinicopathological and cancer-related variables. Patients’ survival rates were calculated with the help of the Kaplan-Meier method, where the log-rank test was used to examine the significance of the differences in the survival rate. The Cox proportional hazards regression model was used to examine the independent prognostic factors. The ImageJ software^1^ was used to analyze cell activity and protein expression. We evaluated it by using unpaired Student’s *t*-test for the comparison of two samples and using a one-way ANOVA test for the comparison of more than two samples. Each experiment was repeated at least three times, and the quantitative data were presented as mean ± standard deviations (SD). α was set at 0.05, and for all statistical analyzes, *P* values less than 0.05 were considered statistically significant.

## Results

### p-JNK Expression in Human Breast Cancer and Survival in Breast Cancer Patients

#### p-JNK Expression in Human Breast Cancer and Adjacent Normal Breast Tissues

**FIGURE 1:**
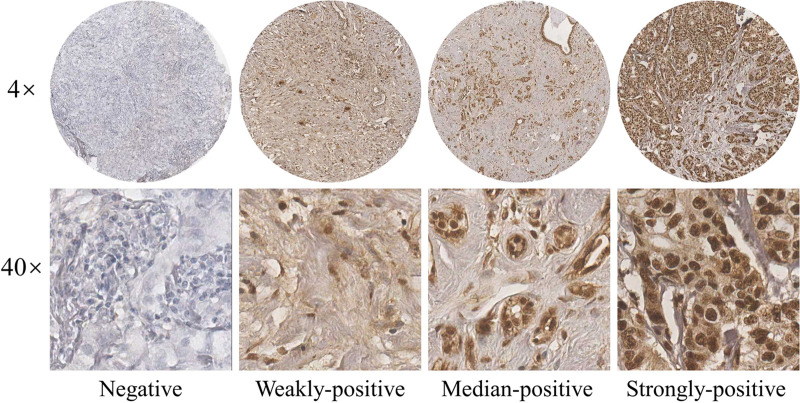
Expression of p-JNK in human breast cancer tissues.

We stained 104 human breast cancer specimens and 65 human adjacent normal breast tissue for p-JNK expression by immunohistochemistry. In 104 human breast cancer specimens, 36 patient samples (34.6%, 36/104) were observed to negative or weakly-positive, and 68 patient samples (65.4%, 68/104) were observed to median-positive or strongly-positive. In 65 human adjacent normal breast tissues, 34 patient samples (52.3%, 34/65) were observed to negative or weakly-positive, and 31 patient samples (47.7%, 31/104) were observed to median-positive or strongly-positive (see Figure 1).

#### Demographic and Clinicopathologic Characteristics of Patients

Table 1 shows the clinicopathological characteristics of breast cancer patients included in the current study. Though both males and females are susceptible to breast cancer, all patients in our study were females (median age = 46 years, 27 to 73 years old). Among all patients, 98 of them were married (94.2%). A majority of the patients did not have a family history of cancer (*n* = 80; 76.9%). Patients’ body mass index (BMI) ranged from 18.08 to 33.73 (median = 23.77). Overall, 64 patients (61.5%) were diagnosed with premenopausal breast cancer, whereas the rest of the patients had a postmenopausal breast cancer diagnosis (*n* = 40; 38.5%). Moreover, 88 patients (84.6%) had mastectomy surgery, while 16 (15.4%) patients received breast conserving surgery. The clinicopathological characteristics were similar between the two groups (see Table 1). Preliminary analyses showed that patients with low p-JNK expression were significantly associated with marital status (χ^2^ = 3.973, *P* = 0.046).

**TABLE 1:** Patients’ demographic and clinicopathologic characteristics.

Parameters	Low p-JNK	High p-JNK	χ^2^	*P* value
Cases (*n*)	36	68		
Age (years)			0.446	0.504
<46	15 (41.67%)	33 (48.53%)		
≥46	21 (58.33%)	35 (51.47%)		
Marital status			3.973	0.046
Married	36 (100.00%)	62 (91.18%)		
Unmarried	0 (0.00%)	6 (8.82%)		
Family history			0.023	0.880
No	28 (77.78%)	52 (76.47%)		
Yes	8 (22.22%)	16 (23.53%)		
BMI			0.042	0.837
<23.77	18 (50.00%)	34 (50.00%)		
≥23.77	18 (50.00%)	34 (50.00%)		
Menopause			0.239	0.625
No	21 (58.33%)	43 (63.24%)		
Yes	15 (41.67%)	25 (36.76%)		
ABO blood type			0.105	0.999
A	10 (27.78%)	18 (26.47%)		
B	12 (33.33%)	22 (32.35%)		
O	9 (25.00%)	19 (27.94%)		
AB	5 (13.89%)	9 (13.24%)		
Tumor site			0.350	0.554
Right	17 (47.22%)	28 (41.18%)		
Left	19 (52.78%)	40 (58.82%)		
US-Primary tumor site			1.327	0.857
Upper outer quadrant	24 (66.67%)	46 (67.65%)		
Lower outer quadrant	4 (11.11%)	4 (5.88%)		
Lower inner quadrant	1 (2.78%)	2 (2.94%)		
Upper inner quadrant	6 (16.67%)	12 (17.65%)		
Central	1 (2.78%)	4 (5.88%)		
US-Tumor size			0.698	0.705
≤2 cm	9 (25.00%)	14 (20.59%)		
>2 and <5 cm	24 (66.67%)	45 (66.18%)		
≥5 cm	3 (8.33%)	9 (13.24%)		
US-LNM			0.003	0.960
No	24 (66.67%)	45 (66.18%)		
Yes	12 (33.33%)	23 (33.82%)		
US-BIRADS			3.654	0.161
4	4 (11.11%)	6 (8.82%)		
5	10 (27.78%)	32 (47.06%)		
6	22 (61.11%)	30 (44.12%)		
Clinical stage				
Clinical T stage			0.218	0.994
T1	5 (13.89%)	8 (11.76%)		
T2	19 (52.78%)	37 (54.41%)		
T3	7 (19.44%)	12 (17.65%)		
T4	5 (13.89%)	11 (16.18%)		
Clinical N stage			2.582	0.630
N0	6 (16.67%)	14 (20.59%)		
N1	15 (41.67%)	18 (26.47%)		
N2	11 (30.56%)	25 (36.76%)		
N3	4 (11.11%)	11 (16.18%)		
Clinical TNM stage			0.262	0.877
I	1 (2.78%)	3 (4.41%)		
II	14 (38.89%)	24 (35.29%)		
III	21 (58.33%)	41 (60.29%)		
Operative time			0.001	0.987
<90	17 (47.22%)	32 (47.06%)		
≥90	19 (52.78%)	36 (52.94%)		
Type of surgery			0.095	0.758
Mastectomy	31 (86.11%)	57 (83.82%)		
Breast-conserving surgery	5 (13.89%)	11 (16.18%)		
Tumor size			0.231	0.891
≤ 2 cm	15 (41.67%)	30 (44.12%)		
>2 and <5 cm	19 (52.78%)	33 (48.53%)		
≥ 5 cm	2 (5.56%)	5 (7.35%)		
Histologic type			0.535	0.465
Ductal	36 (100.00%)	67 (98.53%)		
Lobular	0 (0.00%)	1 (1.47%)		
Histologic grade			4.445	0.108
I	4 (11.11%)	2 (2.94%)		
II	24 (66.67%)	41 (60.29%)		
III	8 (22.22%)	25 (36.76%)		
Pathological TNM classification				
Pathological T stage			0.264	0.992
Tis/T0	1 (2.78%)	3 (4.41%)		
T1	14 (38.89%)	24 (35.29%)		
T2	17 (47.22%)	33 (48.53%)		
T3	2 (5.56%)	4 (5.88%)		
T4	2 (5.56%)	4 (5.88%)		
Pathological N stage			3.468	0.483
N0	12 (33.33%)	18 (26.47%)		
N1	12 (33.33%)	15 (22.06%)		
N2	4 (11.11%)	9 (13.24%)		
N3	8 (22.22%)	26 (38.24%)		
Pathological TNM stage			2.230	0.681
Tis/T0	1 (2.78%)	1 (1.47%)		
I	5 (13.89%)	11 (16.18%)		
II	16 (44.44%)	21 (30.88%)		
III	14 (38.89%)	35 (51.47%)		
Total lymph nodes			0.058	0.810
<23	15 (41.67%)	30 (44.12%)		
≥23	21 (58.33%)	38 (55.88%)		
Positive lymph nodes			1.700	0.192
<2	18 (50.00%)	25 (36.76%)		
≥2	18 (50.00%)	43 (63.24%)		
Total axillary lymph nodes			0.170	0.680
<23	17 (47.22%)	35 (51.47%)		
≥23	19 (52.78%)	33 (48.53%)		
Positive axillary lymph nodes			1.700	0.192
<2	18 (50.00%)	25 (36.76%)		
≥2	18 (50.00%)	43 (63.24%)		
Postoperative chemotherapy			0.421	0.517
No	4 (11.11%)	5 (7.35%)		
Yes	32 (88.89%)	63 (92.65%)		
Postoperative radiotherapy			0.422	0.516
No	10 (27.78%)	15 (22.06%)		
Yes	26 (72.22%)	53 (77.94%)		
Postoperative endocrine therapy			0.514	0.474
No	18 (50.00%)	29 (42.65%)		
Yes	18 (50.00%)	39 (57.35%)		
Postoperative targeted therapy			1.704	0.192
No	22 (61.11%)	50 (73.53%)		
Yes	14 (38.89%)	18 (26.47%)		

#### Univariate and Multivariate Cox Regression Survival Analyses

To analyze the independent prognostic factors, we adopted both the univariate and multivariate Cox proportional-hazards analyses, modeled on time-varying p-JNK expression. Univariate analysis and multivariate analysis were adopted to assess independent prognostic factors (US-LNM, US-BIRADS, clinical T stage, clinical N stage, clinical TNM stage, pre-chemotherapy times, response, tumor size, pathological response, pathological T stage, pathological N stage, pathological TNM stage, positive axillary lymph nodes, postoperative chemotherapy, postoperative endocrine therapy, postoperative targeted therapy, lymph vessel invasion, p-JNK expression). Results of univariate and multivariate Cox regression analyses showed that OS was significantly associated with US-LNM, US-BIRADS, clinical T stage, clinical N stage, clinical TNM stage, pre-chemotherapy times, tumor size, pathological response, pathological T stage, pathological N stage, pathological TNM stage, postoperative chemotherapy, postoperative endocrine therapy, postoperative targeted therapy, lymph vessel invasion, p-JNK expression (see Supplementary Table 1).

#### DFS and OS for the p-JNK Expression of Patients

**FIGURE 2:**
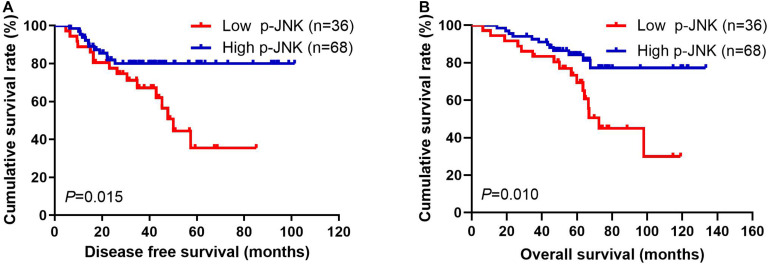
Breast cancer patients’ DFS and OS. **(A)** Patients’ DFS for the p-JNK expression, assessed by the Kaplan-Meier analysis. **(B)** Patients’ of OS for the p-JNK expression, assessed by the Kaplan-Meier analysis.

Patients with high p-JNK expression had prolonged DFS and OS, as indicated by results of univariate analyses (*P* = 0.031, hazard ratio (HR): 0.276, 95% confidence interval (CI): 0.086–0.890 and *P* = 0.004, HR: 0.176, 95% CI: 0.053–0.581, respectively). Furthermore, patients with high p-JNK expression were also related to prolonged DFS and OS by multivariate analysis (*P* = 0.003, HR: 0.214, 95% CI: 0.077–0.597 and *P* = 0.007, HR: 0.194, 95% CI: 0.059–0.633, respectively; Supplementary Table 1). The mean DFS and OS for all enrolled cases with low p-JNK expression were 35.97 months (range from 4.67 to 85.07 months) and 58.40 months (range from 6.43 to 119.03 months), respectively; and the mean DFS and OS for all patients with high p-JNK expression were 38.66 months (range from 6.23 to 101.30 months) and 61.88 months (range from 14.47 to 133.50 months), respectively. The mean DFS and OS times in patients with high p-JNK expression were significantly longer than those in patients with low p-JNK expression by using log-rank methods (χ^2^ = 5.908, *P* = 0.015 and χ^2^ = 6.593, *P* = 0.010, respectively; see Figures 2A,B).

#### Association of Chemotherapy and p-JNK Expression in Patients

All patients had anthracyclines-based and taxanes-based neoadjuvant chemotherapy regimens. Among them, 4 patients received the AC/ACF regimen, 10 patients received the CT/ACT regimen, 53 patients received the AT regimen, 25 patients received TP/ATP regimen, 10 patients received T regimen, whereas 2 patients had other regimens (e.g., ACTP, X regimen). The clinical objective response rate (CR + PR) was 57.7% (60/104), the clinical benefit rate (CR + PR + SD) was 99.0% (103/104), while the non-clinical response rate (SD + PD) was 42.3% (44/104). The Miller-Payne grading (MPG) system was used to evaluate patients’ pathological response, and the grade 1 rate was 8.7% (9/104), the grade 2 rate was 40.4% (42/104), the grade 3 rate was 46.2% (48/104), the grade 4 rate was 1.0% (1/104), and the grade 5 rate was 3.8% (4/104). The pathological response of pCR rate was 5.8% (6/104), and the pathological response of non-pCR rate was 94.2% (98/104). Overall, most patients had postoperative chemotherapy (*n* = 95; 91.3%), only 9 patients (8.7%) did not receive postoperative chemotherapy. And there was no significance difference among these chemotherapy parameters (see Table 2).

**TABLE 2:** The relationship between patients’ chemotherapy and p-JNK expression.

Parameters	Low p-JNK	High p-JNK	χ^2^	*P* value
Cases (*n*)	36	68		
Neoadjuvant Chemotherapy			4.669	0.323
AC/ACF	2 (5.56%)	2 (2.94%)		
CT/ACT	3 (8.33%)	7 (10.29%)		
AT	21 (58.33%)	32 (47.06%)		
TP	8 (22.22%)	13 (19.12%)		
Others	2 (5.56%)	14 (20.59%)		
Pre-chemotherapy times			0.010	0.919
<6	12 (33.33%)	22 (32.35%)		
≥6	24 (66.67%)	46 (67.65%)		
Response			0.701	0.704
PR	20 (55.56%)	40 (58.82%)		
SD	16 (44.44%)	27 (39.71%)		
PD	0 (0.00%)	1 (1.47%)		
Miller and Payne grade			1.542	0.819
1	4 (11.11%)	5 (7.35%)		
2	16 (44.44%)	26 (38.24%)		
3	15 (41.67%)	33 (48.53%)		
4	0 (0.00%)	1 (1.47%)		
5	1 (2.78%)	3 (4.41%)		
Pathological response			1.080	0.299
pCR	0 (0.00%)	2 (2.94%)		
non-pCR	36 (100.00%)	66 (97.06%)		
Postoperative chemotherapy regimen			3.899	0.564
0	4 (11.11%)	5 (7.35%)		
AC/ACF	2 (5.56%)	4 (5.88%)		
CT/ACT	2 (5.56%)	7 (10.29%)		
AT	7 (19.44%)	10 (14.71%)		
TP	14 (38.89%)	19 (27.94%)		
Others	7 (19.44%)	23 (33.82%)		
Postoperative chemotherapy times			1.495	0.221
<4	14 (38.89%)	35 (51.47%)		
≥4	22 (61.11%)	33 (48.53%)		

### Association of Pathology Parameters and p-JNK Expression in Patients

Prior to chemotherapy, patients’ molecular subtypes were diagnosed by core needle biopsy. Analyses showed that 35 patients had Luminal B HER2 negative subtype, 32 patients were triple-negative subtype, 15 patients categorized with HER2-enriched subtype, 14 patients were Luminal B HER2 positive subtype, and 8 patients had Luminal A subtype. Furthermore, 57 patients were Luminal type and 47 patients were non-Luminal type, 32 patients were triple-negative type and 72 patients were non-triple-negative type, 15 patients were HER2 enriched type and 89 patients were non-HER2 enriched type. Among these different molecular subtypes, the results indicated that molecular by HER2 status were significantly different by p-JNK expression (χ^2^ = 4.990, *P* = 0.025, see Table 3).

**TABLE 3:** The relationship between patients’ pathology parameters and p-JNK expression.

Parameters	Low p-JNK	High p-JNK	χ^2^	*P* value
Cases (*n*)	36	68		
Core needle biopsy (Before chemotherapy)				
Molecular subtype (common)			7.156	0.128
Luminal A	2 (5.56%)	6 (8.82%)		
Luminal B HER2 +	2 (5.56%)	12 (17.65%)		
Luminal B HER2-	12 (33.33%)	23 (33.82%)		
HER2 enriched	9 (25.00%)	6 (8.82%)		
Triple negative	11 (30.56%)	21 (30.88%)		
Molecular subtype (by Luminal)			2.387	0.122
Luminal type	16 (44.44%)	41 (60.29%)		
non-Luminal type	20 (55.56%)	27 (39.71%)		
Molecular subtype (by Triple)			0.001	0.973
Triple negative	11 (30.56%)	21 (30.88%)		
non-Triple negative	25 (69.44%)	47 (69.12%)		
Molecular subtype (by HER2)			4.990	0.025
HER2 enriched	9 (25.00%)	6 (8.82%)		
non-HER2 enriched	27 (75.00%)	62 (91.18%)		
ER status			0.784	0.376
Negative	17 (47.22%)	26 (38.24%)		
Positive	19 (52.78%)	42 (61.76%)		
PR status			0.377	0.539
Negative	16 (44.44%)	26 (38.24%)		
Positive	20 (55.56%)	42 (61.76%)		
HER2 status			0.369	0.543
Negative (0– + +)	25 (69.44%)	51 (75.00%)		
Positive (+ + +)	11 (30.56%)	17 (25.00%)		
Ki-67 status			0.317	0.573
Negative (≤ 14%)	8 (22.22%)	12 (17.65%)		
Positive (>14%)	28 (77.78%)	56 (82.35%)		
Postoperative pathology (IHC)				
Molecular subtype (common)			4.287	0.369
Luminal A	5 (13.89%)	12 (17.65%)		
Luminal B HER2 +	1 (2.78%)	8 (11.76%)		
Luminal B HER2-	8 (22.22%)	15 (22.06%)		
HER2 enriched	9 (25.00%)	9 (13.24%)		
Triple negative	13 (36.11%)	24 (35.29%)		
Molecular subtype (by Luminal)			1.495	0.221
Luminal type	14 (38.89%)	35 (51.47%)		
non-Luminal type	22 (61.11%)	33 (48.53%)		
Molecular subtype (by Triple)			0.007	0.934
Triple negative	13 (36.11%)	24 (35.29%)		
non-Triple negative	23 (63.89%)	44 (64.71%)		
Molecular subtype (by HER2)			2.276	0.131
HER2 enriched	9 (25.00%)	9 (13.24%)		
non-HER2 enriched	27 (75.00%)	59 (86.76%)		
ER status			3.286	0.070
Negative	21 (58.33%)	27 (39.71%)		
Positive	15 (41.67%)	41 (60.29%)		
PR status			0.487	0.485
Negative	19 (52.78%)	31 (45.59%)		
Positive	17 (47.22%)	37 (54.41%)		
HER2 status			0.685	0.408
Negative (0– + +)	26 (72.22%)	54 (79.41%)		
Positive (+ + +)	10 (27.78%)	14 (20.59%)		
Ki-67 status			2.350	0.125
Negative (≤ 14%)	16 (44.44%)	20 (29.41%)		
Positive (>14%)	20 (55.56%)	48 (70.59%)		
AR status			0.097	0.755
Negative	32 (88.89%)	59 (86.76%)		
Positive	4 (11.11%)	9 (13.24%)		
CK5/6 status			0.878	0.349
Negative	28 (77.78%)	47 (69.12%)		
Positive	8 (22.22%)	21 (30.88%)		
E-cad status			0.685	0.408
Negative	10 (27.78%)	14 (20.59%)		
Positive	26 (72.22%)	54 (79.41%)		
EGFR status			1.279	0.258
Negative	17 (47.22%)	40 (58.82%)		
Positive	19 (52.78%)	28 (41.18%)		
P53 status			0.009	0.923
Negative	15 (41.67%)	29 (42.65%)		
Positive	21 (58.33%)	39 (57.35%)		
TOP2A status			0.266	0.606
Negative	9 (25.00%)	14 (20.59%)		
Positive	27 (75.00%)	54 (79.41%)		
Lymph vessel invasion			0.002	0.961
Negative	21 (58.33%)	40 (58.82%)		
Positive	15 (41.67%)	28 (41.18%)		
Neural invasion			0.001	0.985
Negative	28 (77.78%)	53 (77.94%)		
Positive	8 (22.22%)	15 (22.06%)		

**FIGURE 3:**
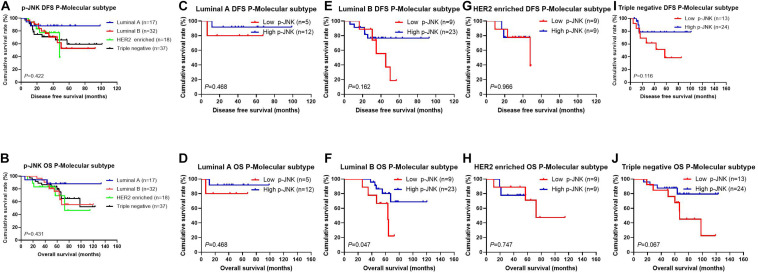
Patients’ DFS and OS for the p-JNK expression, by molecular subtypes. **(A)** Patients’ DFS by molecular subtypes, assessed by the Kaplan-Meier analysis. **(B)** Patients’ OS by molecular subtypes, assessed by the Kaplan-Meier analysis. **(C)** Patients’ DFS by Luminal A subtype, assessed by the Kaplan-Meier analysis. **(D)** Patients’ OS by Luminal A subtype, assessed by the Kaplan-Meier analysis. **(E)** Patients’ DFS by Luminal B subtype, assessed by the Kaplan-Meier analysis. **(F)** Patients’ OS by Luminal B subtype, assessed by the Kaplan-Meier analysis. **(G)** Patients’ DFS by HER2-enriched subtype, assessed by the Kaplan-Meier analysis. **(H)** Patients’ OS by HER2-enriched subtype, assessed by the Kaplan-Meier analysis. **(I)** Patients’ DFS by triple-negative subtype, assessed by the Kaplan-Meier analysis. **(J)** Patients’ OS by triple-negative subtype, assessed by the Kaplan-Meier analysis.

After operation, we also detected different molecular subtypes in patients by postoperative pathology (IHC). The molecular subtypes were shown in Table 3. We divided these molecular subtypes were into four categories: Luminal A, Luminal B, HER2-enriched, and triple-negative. Results of the log-rank test showed that the mean DFS and OS times for patients in molecular subtypes were not significant (χ^2^ = 2.812, *P* = 0.422, see Figure 3A, and χ^2^ = 2.757, *P* = 0.431, see Figure 3B, respectively). We found that mean DFS and OS times in patients with high p-JNK expression were longer than those in patients with low p-JNK expression in Luminal A (49.50 months vs 36.75 months, 68.35 months vs 51.57 months, respectively) and Luminal B molecular subtypes(37.24 months vs 35.48 months, 62.70 months vs 52.90 months, respectively). Whereas mean DFS and OS time in patients with high p-JNK expression was similar to patients with triple-negative molecular subtype (36.98 months vs 37.90 months, 65.44 months vs 65.42 months). However, the mean DFS and OS times in patients with high p-JNK expression were shorter than those in patients with low p-JNK expression in HER2-enriched molecular subtype (32.28 months vs 33.26 months, 41.67 months vs 57.56 months, respectively). Findings showed that the mean OS time in patients with high p-JNK expression was significantly longer than that in patients with low p-JNK expression in the Luminal B molecular subtype (χ^2^ = 3.950, *P* = 0.047) (see Figures 3C–J).

#### Correlation Between Lymph Vessel Invasion and p-JNK Expression in Patients

**FIGURE 4:**
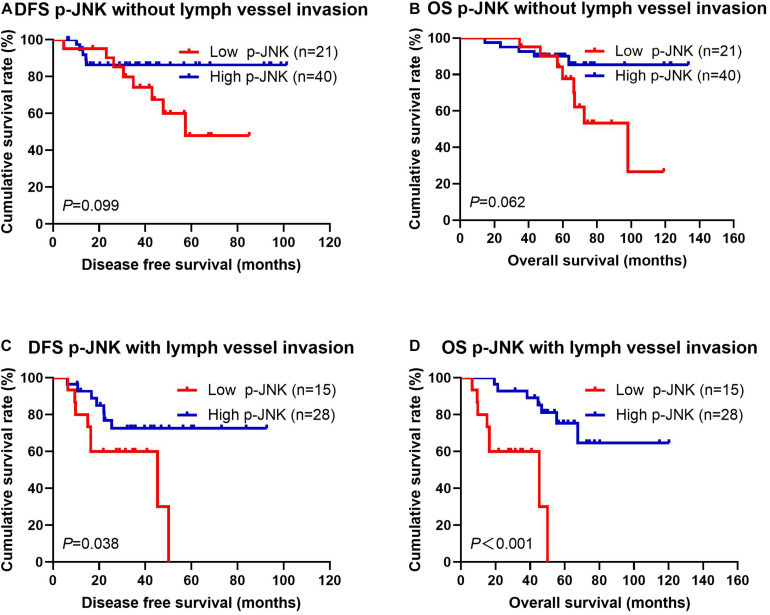
Patients’ DFS and OS by lymph vessel invasion status. **(A)** Patients’ DFS without lymph vessel invasion by p-JNK expression, assessed by the Kaplan-Meier analysis. **(B)** Patients’ OS without lymph vessel invasion by p-JNK expression, assessed by the Kaplan-Meier analysis. **(C)** Patients’ DFS with lymph vessel invasion by p-JNK expression, assessed by the Kaplan-Meier analysis. **(D)** Patients’ OS with lymph vessel invasion by p-JNK expression, assessed by the Kaplan-Meier analysis.

According to univariate and multivariate analyses, the lymph vessel invasion was a significant prognostic factor (see Supplementary Table 1). To further investigate the prognostic efficiency of p-JNK expression, we analyzed the lymph vessel invasion by p-JNK expression. The lymph vessel invasion status was divided into two categories: without lymph vessel invasion and with lymph vessel invasion. The mean DFS and OS in patients without lymph vessel invasion were 43.13 months and 65.61 months with high p-JNK expression; 39.42 months and 64.76 months with low p-JNK expression, respectively. The results indicated that the mean DFS and OS times in patients with high p-JNK expression by the log-rank test were longer than those in patients with low p-JNK expression without lymph vessel invasion (χ^2^ = 2.715, *P* = 0.099 and χ^2^ = 3.477, *P* = 0.062, respectively; Figures 4A,B). The mean DFS and OS times in patients with lymph vessel invasion were 25.96 months and 48.31 months with low p-JNK expression; 37.57 months and 57.77 months with high p-JNK expression, respectively. The results indicated that the mean DFS and OS times in patients with high p-JNK expression by the log-rank test were longer than those in patients with low p-JNK expression with lymph vessel invasion (χ^2^ = 4.302, *P* = 0.038 and χ^2^ = 14.020, *P*<0.001, respectively; Figures 4C,D). The results also indicated that patients with lymph vessel invasion and low p-JNK expression survived shorter, and had worse prognose (see Figure 4).

#### Correlation Between p-JNK Expression and Side Effects of Chemotherapy

In this study, hematologic and gastrointestinal reactions were found to be common toxicities after NACT. The National Cancer Institute-Common Toxicity Criteria (NCI-CTC) was adopted to evaluate and analyze any potential side effects of NACT (Eisenhauer et al., 2009). To further evaluate side effects of NACT, p-JNKs were utilized in our study. Findings indicated that before-NACT p-JNK expressions were not significantly related to toxicities of enrolled patients, except mouth ulcers (see Table 4). None of the patients enrolled in the study suffered from chemotherapy-related deaths.

**TABLE 4:** Correlation between p-JNK expression and patients’ chemotherapy side effects.

Parameters	Low p-JNK	High p-JNK	χ^2^	*P* value
Cases (*n*)	36	68		
Decreased appetite			0.004	0.949
No	6 (16.67%)	11 (16.18%)		
Yes	30 (83.33%)	57 (83.82%)		
Nausea			0.017	0.897
No	4 (11.11%)	7 (10.29%)		
Yes	32 (88.89%)	61 (89.71%)		
Vomiting			0.487	0.485
No	19 (52.78%)	31 (45.59%)		
Yes	17 (47.22%)	37 (54.41%)		
Diarrhea			0.225	0.635
No	33 (91.67%)	64 (94.12%)		
Yes	3 (8.33%)	4 (5.88%)		
Mouth ulcers			3.851	0.049
No	34 (94.44%)	68 (100.00%)		
Yes	2 (5.56%)	0 (0.00%)		
Alopecia			0.446	0.504
No	15 (41.67%)	33 (48.53%)		
Yes	21 (58.33%)	35 (51.47%)		
Peripheral neurotoxicity			0.004	0.949
No	30 (83.33%)	57 (83.82%)		
Yes	6 (16.67%)	11 (16.18%)		
Anemia			2.781	0.095
Grade 0	15 (41.67%)	40 (58.82%)		
Grade 1-2	21 (58.33%)	28 (41.18%)		
Grade 3-4	0 (0.00%)	0 (0.00%)		
Leukopenia			4.347	0.113
Grade 0	11 (30.56%)	13 (19.12%)		
Grade 1-2	14 (38.89%)	41 (60.29%)		
Grade 3-4	11 (30.56%)	14 (20.59%)		
Neutropenia			0.979	0.613
Grade 0	7 (19.44%)	13 (19.12%)		
Grade 1-2	12 (33.33%)	29 (42.65%)		
Grade 3-4	17 (47.22%)	26 (38.24%)		
Thrombocytopenia			0.099	0.752
Grade 0	28 (77.78%)	51 (75.00%)		
Grade 1-2	8 (22.22%)	17 (25.00%)		
Grade 3-4	0 (0.00%)	0 (0.00%)		
Gastrointestinal reaction			1.910	0.385
Grade 0	4 (11.11%)	8 (11.76%)		
Grade 1-2	31 (86.11%)	60 (88.24%)		
Grade 3-4	1 (2.78%)	0 (0.00%)		
Myelosuppression			0.152	0.927
Grade 0	5 (13.89%)	10 (14.71%)		
Grade 1-2	10 (27.78%)	21 (30.88%)		
Grade 3-4	21 (58.33%)	37 (54.41%)		
Hepatic dysfunction			0.131	0.717
Grade 0	22 (61.11%)	44 (64.71%)		
Grade 1-2	14 (38.89%)	24 (35.29%)		
Grade 3-4	0 (0.00%)	0 (0.00%)		

### Anisomycin Activates JNK and Induces Apoptosis

#### Anisomycin Induces Apoptosis in Breast Cancer

**FIGURE 5:**
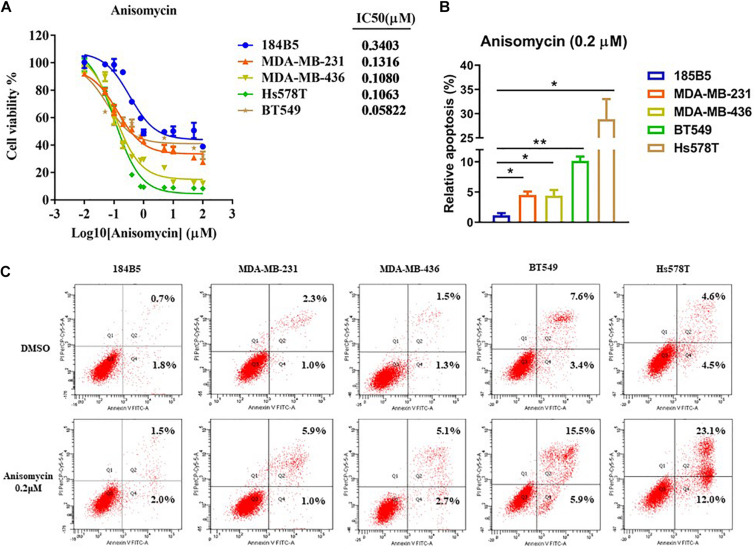
Apoptosis induced by anisomycin in human breast cancer. **(A)** CCK-8 detected IC50 of human mammary epithelial cell line (184B5) and human breast cancer cell lines (MDA-MB-231, MDA-MB-436, BT549, and Hs578T). **(B)** Prior to flow cytometry, human mammary epithelial cell and breast cancer cells were conditioned with 0 μM or 0.2 μM anisomycin for 48 h. **(C)** Flow cytometry analyzed apoptosis of 184B5, MDA-MB-231, MDA-MB-436, BT549, and Hs578T.

To assess the cytotoxicity of anisomycin on breast cancer cells, cells were treated with anisomycin at the following concentrations: 100, 50, 10, 5, 1, 0.8, 0.4, 0.2, 0.1, 0.05, 0.01, 0 μM for 48h. Cell viability was analyzed by CCK-8 assay. The data demonstrated that the IC50 value of human mammary epithelial cell line (184B5) was 0.3403μM. The IC50 value of human breast cancer cell lines (MDA-MB-231, MDA-MB-436, BT549, Hs578T) were 0.1316, 0.1080, 0.0582, 0.1063 μM, respectively (see Figure 5A). And 0.2 μM was chosen as the suitable concentration in the subsequent experiments.

**FIGURE 6:**
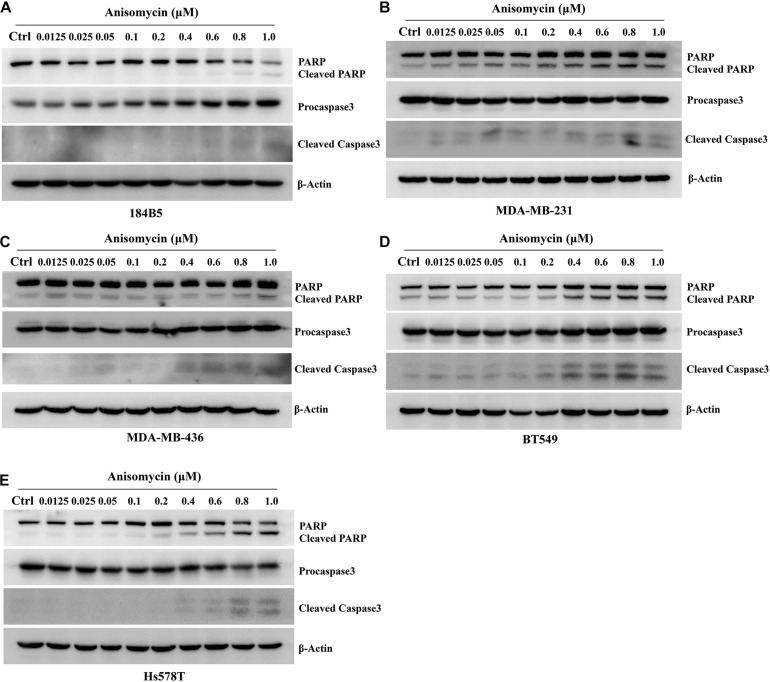
Apoptosis induced by anisomycin in human mammary epithelial cell line and human breast cancer cell lines. **(A–E)** 184B5 **(A)**, MDA-MB-231 **(B)**, MDA-MB-436 **(C)**, BT549 **(D)**, and Hs578T **(E)** cells were treated with indicated concentration of anisomycin. After being treated with anixomycin for 48 h, western blot was adopted to examine PARP, procaspase3, cleaved caspase-3, and β-actin expressions.

We next investigate the effect of anisomycin on apoptosis. The breast cancer cells were treated with 0 or 0.2 μM anisomycin for 48h, and then detected by flow cytometry. As expected, anisomycin can induce apoptosis in breast cancer cell lines. Moreover, human mammary epithelial cell line (184B5) treated with anisomycin showed reduced apoptosis compared with human breast cancer cell lines (Figures 5B,C). And we also detected the expression of PARP and caspase-3 by western blot after adding different concentrations of anisomycin, and found that breast cancer cells with higher concentration anisomycin showed significantly increased expression of both cleaved caspase-3 and cleaved PARP than those with lower concentration anisomycin (see Figures 6A–E).

#### Anisomycin Activates JNK and Inhibits the Activation of STAT3

**FIGURE 7:**
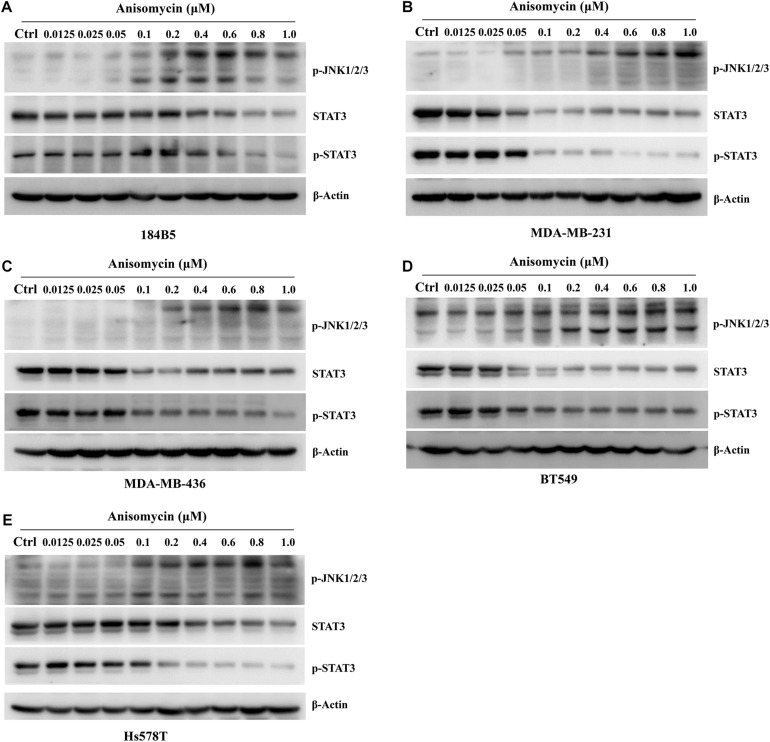
Anisomycin activated JNK and inhibited the activation of STAT3. **(A–E)** 184B5 **(A)**, MDA-MB-231 **(B)**, MDA-MB-436 **(C)**, BT549 **(D)**, and Hs578T **(E)** cells were conditioned with anisomycin, concentration as indicated. After being treated with anixomycin for 48 h, western blot was adopted to examine STAT3, p-STAT3, p-JNK1/2/3, and -actin expression.

Anisomycin was known to induce cell death and JNK activation was required for Anisomycin induced apoptosis (Zhou et al., 2019; Zhang et al., 2020). We examined the p-JNK expression by western blot after the treatment of anisomycin in human breast cancer cell lines (MDA-MB-231, MDA-MB-436, BT549, Hs578T). The results indicated that anisomycin was a potent activator of JNK in human breast cancer cell lines. And anisomycin would present a better option for delineating the effects of p-JNK expression (Figure 6). Previous studies showed that STAT3 activation is mediated by the combined action of JAK, SRC, c-ABL, and JNKs (Chakraborty et al., 2017). To further investigate the mechanism of anisomycin inducing apoptosis, we used western blot to detect the STAT3/p-STAT3 expression after the treatment of anisomycin in human breast cancer cell lines (i.e., MDA-MB-231, MDA-MB-436, BT549, and Hs578T). The results indicated that anisomycin was a potent inhibitor of STAT3 in human breast cancer cell lines. In other words, findings showed that anisomycin induced apoptosis by activating JNKs and restraining the activation of STAT3 (see Figure 7).

## Discussion

Approximately 2.1 million people will be diagnosed with breast cancer in 2018, effectively making the ever-growing breast cancer population even larger (Ferlay et al., 2018). Breast cancer is a heterogeneous disease that can be caused by many signaling pathways that are responsible for cell proliferation and cell apoptosis, such as PI3K/AKT/mTOR, JAK/STAT, PTEN/AKT/MDM2/p53, and AKT/NF-κB signaling pathway (Wan et al., 2019; Lee et al., 2020; Martínez-Rodríguez et al., 2020; Narayanankutty, 2020; Nunnery and Mayer, 2020). Subsequently, due to genetic variability, different breast patients often exhibit varied susceptibility to different signaling pathways. Hence, providing patients with individualized treatments is of critical importance in helping patients better cope with cancer care and management. However, although many of potential targets of treatments exist in breast cancer cells, the mechanism of these anti-cancer targets is still not clear.

MAPKs can be activated via a kinase signaling cascade in which a MAP3K activates a MAP2K, and in turn activates a MAPK. JNKs are subfamilies of MAPKs (Aikin et al., 2020). There are three proteins of JNKs in mammals: JNK1, JNK2 and JNK3, and they are encoded by three distinctive genes jnk1 (Mapk8), jnk2 (Mapk9), and jnk3 (Mapk10), respectively. Although JNK1 and JNK2 are expressed in most tissues, JNK3 expression is mainly limited in brain, heart and testis (Kumar et al., 2015). Upon activation by the upstream MAP2Ks, JNKs phosphorylate can activate a considerable number of nuclear and non-nuclear proteins, such as the transcription factor activator protein-1 (AP-1) (Lee et al., 2020). And these proteins control a diversity of cellular responses, such as cell growth, cell proliferation, cell differentiation, cell survival and cell death. The aberrant expression and activation of JNKs are found in many cancer cell lines and tissue samples (Mohebali et al., 2020; Qu et al., 2020). In primary hepatocellular carcinoma (HCC), compared with the non-neoplastic lesions, the activation of JNK1 in tumor size was significantly increased, and absence of JNK1 impaired hepatocyte proliferation and tumor formation (Chang et al., 2009; Gao et al., 2019). In mice with DEN induced liver cancer, the levels of activated JNK (p-JNK) were decreased by D-JNKI-1 injection for inhibited three months in the treatment group, whereas the levels of p-JNK was continuously expressed high (Davoli et al., 2014). However, the p-JNK was rarely studied, especially in breast cancer patients who had NACT.

In order to study the p-JNK expression in breast cancer patients’ tissues, we stained 104 human breast cancer specimens and 65 human adjacent normal breast tissue for p-JNK expression by immunohistochemistry. The results indicated that 65.4% of reviewed cases were observed to be strongly-positive in human breast cancer specimens, however, 47.7% of the cases were observed to be strongly-positive in human adjacent normal breast tissues. To further analyze the relationship between the expression of p-JNK and the prognosis of breast cancer patients, we used the univariate and multivariate Cox proportional-hazards models to evaluate relevant independent prognostic factors. The results proved that p-JNK expression was an independent prognostic factor of DFS and OS. Patients with high p-JNK expression related to prolonged DFS and OS than those patients with low p-JNK expression by log-rank methods.

Anisomycin was a potent protein synthesis inhibitor, and was widely used as an agonist for p38 MAPK and JNK. It is generally known that anisomycin induced apoptosis in a variety of cell types through the activation of the p38 MAPK and the JNK pathway (Li et al., 2018; Yang et al., 2020). In our study, we found that anisomycin could significantly inhibit cell proliferation and promote cell apoptosis. The IC50 of human breast cancer cell lines were higher than human mammary epithelial cell lines. We also used western blot to detect the expression of cleaved caspase-3 and cleaved PARP, and found that the expression of cleaved caspase-3 and cleaved PARP were significantly higher by high concentration anisomycin. Some studies have proved that anisomycin was a JNK activator, and also increased phospho-JNK (Guan et al., 2020; Yoon et al., 2020). Hence, we used western blot to detect the p-JNK expression by anisomycin, and the results indicated that anisomycin was a potent activator of JNK in human breast cancer cell lines. Combined with cleaved caspase-3 and cleaved PARP, we found that the p-JNK expression is positively associated with the cleaved caspase-3 and cleaved PARP by anisomycin.

JNKs, as subtypes of MAPKs, mediate the stress-dependent serine phosphorylation of STAT3 (Chen et al., 2013). JNKs were activated by UV or anisomycin or by their upstream kinase MEKK1 phosphorylation STAT3 *in vitro* (Nikaido et al., 2018). STAT3 was also phosphorylated by cotransfection of JNKs with MEKK1 *in vivo* (Ni et al., 2003). And the experiments confirmed that the major phosphorylation site of STAT3 by JNKs was identified to be Ser-727 *in vitro* (Lim and Cao, 1999; Schuringa et al., 2000; Miyazaki et al., 2008). The STAT family of transcription factors integrated cytokine and growth factor signaling to transcriptionally regulate a diverse array of cellular processes (Corry et al., 2020). STAT3 had become one of the common investigated oncogenic transcription factors and was associated with cell proliferation, differentiation, progression, metastasis and chemoresistance (Qin et al., 2019). STAT3 was activated via the phosphorylation of Y705 by cytoplasmic non-receptor tyrosine kinases (Johnson et al., 2018).

One study showed that NSC-743380 modulates functions of multipathways, including activating MAP kinase JNK and inhibiting JAK/STAT3 pathway, had potential *in vitro* and *in vivo* antitumor activities (Guo et al., 2011). In our study, we used western blot to detect the expression of p-JNK and p-STAT3 by anisomycin, and the results indicated that the protein expression of p-JNK increased with anisomycin concentration, whereas the protein expression of p-STAT3 decreased with anisomycin concentration, and they had the inverse correlation relations.

Meanwhile, as results of univariate and multivariate Cox regression analyses suggested, occupation, US-LNM, US-BIRADS, clinical T stage, clinical N stage, clinical TNM stage, tumor size, pathological T stage, postoperative endocrine therapy, postoperative targeted therapy, and lymph vessel invasion were significant prognostic factors in predicting patients’ improved DFS and OS. Lymph vessel invasion is thought to play an important role in tumor metastasis, and acts as the modulation of antitumor immune responses (David Nathanson et al., 2020; Testa et al., 2020; Wang et al., 2020). The tumor angiogenesis and its indicative vascular density are closely related to the prognosis of breast cancer (Huang et al., 2020; Koch et al., 2020). In our research, the results also indicated that lymph vessel invasion was the significant prognostic factor, and the patients with lymph vessel invasion and low JNK expression survived shorter. Although the molecular subtypes were not significant prognostic factors by univariate and multivariate analyses, the mean DFS and OS times in patients with high JNK expression were longer than those in patients with low JNK expression in molecular subtypes, especially in Luminal B molecular subtype.

### Limitations

There are several limitations in this study. Firstly, we only examined the anisomycin in breast cancer cells, but not in patients with breast cancer. Future prospective and randomized controlled trials can further extend our research on anisomycin in breast cancer patients. Secondly, this study was a retrospective single-center study and the number of enrolled patients was not large. Future study can benefit from having more patients enrolled, and adopting a multicenter study design. Furthermore, having a large sample size can also help improve rigor in subgroup analyses.

## Conclusion

In summary, anisomycin was a potent activator of JNK in human breast cancer cell lines, and can present a better option for delineating the effects of p-JNK expression. p-JNK expression, on the other hand, is a significant and effective prognostic predictor of survival time in breast cancer patients receiving NACT. Taken together, we demonstrated that p-JNKs are independent prognostic markers for breast cancer patients. Further research should pour more attention into understanding the functions of p-JNKs and p-STAT3 in breast cancer formation and treatment, to help clinicians and scholars better understand breast cancer development and progression, and in turn, offer new insights and novel solutions that could guide future breast cancer research.

## Data Availability Statement

The raw data supporting the conclusions of this article will be made available by the authors, without undue reservation.

## Ethics Statement

This study was approved by the Ethics Committee of the Cancer Hospital Chinese Academy of Medical Sciences. The patients/participants provided their written informed consent to participate in this study.

## Author Contributions

YF and JW: conceptualization and funding acquisition. AL and ZL: project administration and supervision. SL: validation. XK and XW: visualization. LC and XZ: writing-original draft. LC, XZ, and ZS: writing-review editing. The authors read and approved the final manuscript. All authors contributed to the article and approved the submitted version.

## Conflict of Interest

The authors declare that the research was conducted in the absence of any commercial or financial relationships that could be construed as a potential conflict of interest.

## Footnotes

1 http://rsb.info.nih.gov/ij/
