# Supplementary material for: The Prognostic Significance of Anisomycin-Activated Phospho-c-Jun NH2-Terminal Kinase (p-JNK) in Predicting Breast Cancer Patients’ Survival Time
Source: Front Cell Dev Biol. 2021 Mar 9;9:656693. doi: 10.3389/fcell.2021.656693 (PMC7985183; doi:10.3389/fcell.2021.656693)
Supplement: Supplementary file 1 [file Table_1.DOCX]

Table 1 Univariate and multivariate Cox regression survival analyses of the p-JNK expression for the prediction of DFS and OS in patients with breast cancer

|  |  | DFS |  |  |  | OS |  |  |
| --- | --- | --- | --- | --- | --- | --- | --- | --- |
|  | Univariate analysis |  | Multivariate analysis |  | Univariate analysis |  | Multivariate analysis |  |
| Parameters | Hazard ratio (95%CI) | P value | Hazard ratio (95%CI) | P value | Hazard ratio (95%CI) | P value | Hazard ratio (95%CI) | P value |
| Age (years) |  | 0.461 |  |  |  | 0.053 |  |  |
| ＜46 | 1(reference) |  |  |  | 1(reference) |  |  |  |
| ≥46 | 1.689(0.419-6.802) |  |  |  | 3.653(0.982-13.595) |  |  |  |
| Marital status |  | 0.262 |  |  |  | 0.425 |  |  |
| Married | 1(reference) |  |  |  | 1(reference) |  |  |  |
| Unmarried | 0.512(0.159-1.648) |  |  |  | 0.622(0.193-1.997) |  |  |  |
| Family history |  | 0.226 |  |  |  | 0.059 |  |  |
| No | 1(reference) |  |  |  | 1(reference) |  |  |  |
| Yes | 3.400(0.468-24.674) |  |  |  | 3.771(0.949-14.990) |  |  |  |
| BMI |  | 0.407 |  |  |  | 0.283 |  |  |
| ＜23.77 | 1(reference) |  |  |  | 1(reference) |  |  |  |
| ≥23.77 | 1.465(0.593-3.619) |  |  |  | 1.546(0.698-3.424) |  |  |  |
| Menopause |  | 0.512 |  |  |  | 0.189 |  |  |
| No | 1(reference) |  |  |  | 1(reference) |  |  |  |
| Yes | 1.704(0.346-8.390) |  |  |  | 2.457(0.642-9.397) |  |  |  |
| ABO blood type |  | 0.456 |  |  |  | 0.095 |  |  |
| A | 1(reference) |  |  |  | 1(reference) |  |  |  |
| B | 1.664(0.802-3.453) |  |  |  | 2.382(1.094-5.184) |  |  |  |
| O | 1.054(0.490-2.264) |  |  |  | 1.268(0.585-2.750) |  |  |  |
| AB | 1.218(0.464-3.200) |  |  |  | 2.477(0.914-6.714) |  |  |  |
| Tumor site |  | 0.400 |  |  |  | 0.855 |  |  |
| Right | 1(reference) |  |  |  | 1(reference) |  |  |  |
| Left | 1.309(0.700-2.448) |  |  |  | 1.061(0.562-2.002) |  |  |  |
| US-Primary tumor site |  | 0.876 |  |  |  | 0.520 |  |  |
| Upper outer quadrant | 1(reference) |  |  |  | 1(reference) |  |  |  |
| Lower outer quadrant | 1.052(0.295-3.756) |  |  |  | 0.656(0.194-2.217) |  |  |  |
| Lower inner quadrant | 1.419(0.153-13.165) |  |  |  | 1.764(0.458-12.582) |  |  |  |
| Upper inner quadrant | 0.976(0.448-2.128) |  |  |  | 1.253(0.553-2.838) |  |  |  |
| Central | 0.396(0.071-2.204) |  |  |  | 0.438(0.077-2.507) |  |  |  |
| US-Tumor size |  | 0.672 |  |  |  | 0.273 |  |  |
| ≤2cm | 1(reference) |  |  |  | 1(reference) |  |  |  |
| ＞2 and ＜5cm | 1.377(0.659-2.879) |  |  |  | 1.243(0.595-2.596) |  |  |  |
| ≥5cm | 1.526(0.494-4.716) |  |  |  | 2.392(0.790-7.236) |  |  |  |
| US-LNM |  | 0.034 |  | 0.012 |  | ＜0.001 |  | ＜0.001 |
| No | 1(reference) |  | 1(reference) |  | 1(reference) |  | 1(reference) |  |
| Yes | 3.293(1.092-9.930) |  | 1.956(1.158-3.304) |  | 6.743(2.531-17.967) |  | 3.503(1.876-6.542) |  |
| US-BIRADS |  | 0.036 |  | 0.001 |  | 0.002 |  | 0.005 |
| 4 | 1(reference) |  | 1(reference) |  | 1(reference) |  | 1(reference) |  |
| 5 | 2.003(1.096-8.863) |  | 1.183(1.039-2.116) |  | 1.603(1.170-5.725) |  | 1.420(1.082-2.159) |  |
| 6 | 2.650(1.210-9.321) |  | 1.894(1.216-2.245) |  | 1.580(1.198-4.488) |  | 1.530(1.108-2.599) |  |
| Clinical stage |  |  |  |  |  |  |  |  |
| Clinical T stage |  | 0.001 |  | 0.002 |  | 0.002 |  | 0.005 |
| T1 | 1(reference) |  | 1(reference) |  | 1(reference) |  | 1(reference) |  |
| T2 | 1.381(1.270-7.710) |  | 1.147(1.013-5.520) |  | 1.467(1.398-7.338) |  | 1.262(1.098-3.602) |  |
| T3 | 1.798(1.301-18.028) |  | 1.339(1.040-2.843) |  | 1.663(1.300-5.965) |  | 1.771(1.281-3.717) |  |
| T4 | 1.967(1.367-12.232) |  | 1.511(1.065-6.004) |  | 1.965(1.528-24.846) |  | 2.113(1.764-5.738) |  |
| Clinical N stage |  | 0.009 |  | 0.022 |  | 0.027 |  | 0.041 |
| N0 | 1(reference) |  | 1(reference) |  | 1(reference) |  | 1(reference) |  |
| N1 | 2.270(1.135-13.682) |  | 1.238(1.100-3.299) |  | 3.191(1.115-22.584) |  | 1.764(1.370-4.432) |  |
| N2 | 3.191(1.259-22.584) |  | 1.450(1.050-3.736) |  | 3.617(2.104-25.349) |  | 3.526(1.442-12.736) |  |
| N3 | 3.617(1.971-25.349) |  | 1.699(1.115-4.901) |  | 3.867(2.116-27.871) |  | 6.966(1.701-28.534) |  |
| Clinical TNM stage |  | 0.016 |  | 0.005 |  | 0.004 |  | 0.012 |
| I | 1(reference) |  | 1(reference) |  | 1(reference) |  | 1(reference) |  |
| II | 1.249(1.042-5.617) |  | 2.812(1.203-9.516) |  | 3.697(1.100-18.085) |  | 1.934(1.054-9.492) |  |
| III | 1.952(1.150-7.588) |  | 4.555(1.347-15.412) |  | 4.218(1.230-14.462) |  | 2.974(1.728-29.980) |  |
| Neoadjuvant Chemotherapy |  | 0.673 |  |  |  | 0.206 |  |  |
| AC/ACF | 1(reference) |  |  |  | 1(reference) |  |  |  |
| CT/ACT | 1.249(0.286-5.463) |  |  |  | 0.705(0.157-3.162) |  |  |  |
| AT | 0.935(0.259-3.373) |  |  |  | 0.559(0.144-2.166) |  |  |  |
| TP | 0.564(0.125-2.533) |  |  |  | 0.693(0.136-3.537) |  |  |  |
| Others | 0.665(0.171-2.593) |  |  |  | 0.254(0.060-1.081) |  |  |  |
| Pre-chemotherapy times |  | 0.032 |  | 0.020 |  | 0.008 |  | 0.007 |
| ＜6 | 1(reference) |  | 1(reference) |  | 1(reference) |  | 1(reference) |  |
| ≥6 | 1.967(1.060-3.648) |  | 1.884(1.107-3.207) |  | 2.088(1.215-3.588) |  | 2.107(1.229-3.612) |  |
| Response |  | 0.013 |  | 0.001 |  | 0.570 |  |  |
| PR | 1(reference) |  | 1(reference) |  | 1(reference) |  |  |  |
| SD+PD | 1.536(0.318-5.307) |  | 1.791(0.476-3.315) |  | 1.395(0.432-4.502) |  |  |  |
| Operative time |  | 0.107 |  |  |  | 0.214 |  |  |
| ＜90 | 1(reference) |  |  |  | 1(reference) |  |  |  |
| ≥90 | 0.615(0.340-1.112) |  |  |  | 0.696(0.392-1.233) |  |  |  |
| Type of surgery |  | 0.555 |  |  |  | 0.951 |  |  |
| Mastectomy | 1(reference) |  |  |  | 1(reference) |  |  |  |
| Breast-conserving surgery | 1.620(0.326-8.044) |  |  |  | 1.049(0.228-4.832) |  |  |  |
| Tumor size |  | ＜0.001 |  | ＜0.001 |  | ＜0.001 |  | ＜0.001 |
| ≤2cm | 1(reference) |  | 1(reference) |  | 1(reference) |  | 1(reference) |  |
| ＞2 and ＜5cm | 7.682(1.840-32.082) |  | 7.797(3.116-19.511) |  | 4.298(1.022-18.085) |  | 3.696(1.438-9.498) |  |
| ≥5cm | 12.386(4.163-36.854) |  | 10.326(3.849-27.698) |  | 15.784(1.571-58.550) |  | 11.818(4.081-34.226) |  |
| Miller and Payne grade |  | 0.473 |  |  |  | 0.082 |  |  |
| 1 | 1(reference) |  |  |  | 1(reference) |  |  |  |
| 2 | 0.203(0.026-1.610) |  |  |  | 0.071(0.009-0.551) |  |  |  |
| 3 | 0.207(0.027-1.593) |  |  |  | 0.050(0.006-0.388) |  |  |  |
| 4 | 0.044(0.001-1.798) |  |  |  | 0.008(0.000-7.298) |  |  |  |
| 5 | 0.012(0.001-1.504) |  |  |  | 0.001(0.000-3.938) |  |  |  |
| Histologic type |  | 0.296 |  |  |  | 0.660 |  |  |
| Ductal | 1(reference) |  |  |  | 1(reference) |  |  |  |
| Lobular | 2.123(0.113-9.765) |  |  |  | 2.520(0.041-154.770) |  |  |  |
| Pathological response |  | 0.008 |  | 0.018 |  | 0.024 |  | 0.005 |
| pCR | 1(reference) |  | 1(reference) |  | 1(reference) |  | 1(reference) |  |
| non-pCR | 0.044(0.005-0.436) |  | 0.074(0.009-0.645) |  | 0.049(0.004-0.676) |  | 0.036(0.004-0.365) |  |
| Histologic grade |  | 0.367 |  |  |  | 0.089 |  |  |
| I | 1(reference) |  |  |  | 1(reference) |  |  |  |
| II | 6.439(0.457-90.749) |  |  |  | 2.195(0.501-9.623) |  |  |  |
| III | 3.952(0.269-58.081) |  |  |  | 2.978(1.065-15.785) |  |  |  |
| Pathological TNM classification |  |  |  |  |  |  |  |  |
| Pathological T stage |  | 0.016 |  | 0.015 |  | 0.008 |  | 0.006 |
| Tis/T0 | 1(reference) |  | 1(reference) |  | 1(reference) |  | 1(reference) |  |
| T1 | 1.868(1.186-6.420) |  | 1.521(1.025-3.924) |  | 1.621(1.253-6.821) |  | 1.338(1.063-1.829) |  |
| T2 | 2.705(1.163-43.505) |  | 2.210(1.215-4.272) |  | 1.896(1.013-9.355) |  | 2.305(1.677-7.850) |  |
| T3 | 3.794(1.535-60.130) |  | 3.085(1.490-24.593) |  | 2.552(1.318-15.071) |  | 2.516(1.505-12.537) |  |
| T4 | 4.289(3.592-71.641) |  | 6.226(1.308-25.709) |  | 2.852(1.487-16.004) |  | 4.137(1.112-15.400) |  |
| Pathological N stage |  | 0.011 |  | 0.032 |  | 0.013 |  | 0.008 |
| N0 | 1(reference) |  | 1(reference) |  | 1(reference) |  | 1(reference) |  |
| N1 | 1.101(1.016-1.654) |  | 1.212(1.011-1.615) |  | 1.454(1.047-2.412) |  | 1.575(1.128-2.212) |  |
| N2 | 1.289(1.048-1.738) |  | 1.263(1.042-1.668) |  | 1.706(1.383-2.710) |  | 1.890(1.375-2.112) |  |
| N3 | 1.604(1.212-1.717) |  | 2.088(1.085-4.018) |  | 2.251(1.189-4.259) |  | 2.426(1.284-4.581) |  |
| Pathological TNM stage |  | 0.001 |  | 0.001 |  | 0.002 |  | 0.001 |
| Tis/T0+I | 1(reference) |  | 1(reference) |  | 1(reference) |  | 1(reference) |  |
| II | 1.124(1.038-1.401) |  | 1.118(1.037-1.373) |  | 1.120(1.026-1.560) |  | 1.144(1.033-1.638) |  |
| III | 1.960(1.195-4.719) |  | 1.730(1.160-3.336) |  | 1.716(1.152-3.366) |  | 1.923(1.212-4.016) |  |
| Total lymph nodes |  | 0.416 |  |  |  | 0.724 |  |  |
| ＜23 | 1(reference) |  |  |  | 1(reference) |  |  |  |
| ≥23 | 1.648(0.495-5.489) |  |  |  | 1.093(0.667-1.793) |  |  |  |
| Positive lymph nodes |  | 0.096 |  |  |  | 0.081 |  |  |
| ＜2 | 1(reference) |  |  |  | 1(reference) |  |  |  |
| ≥2 | 1.750(0.906-3.380) |  |  |  | 3.621(0.853-15.374) |  |  |  |
| Total axillary lymph nodes |  | 0.378 |  |  |  | 0.339 |  |  |
| ＜23 | 1(reference) |  |  |  | 1(reference) |  |  |  |
| ≥23 | 1.254(0.758-2.076) |  |  |  | 1.277(0.773-2.110) |  |  |  |
| Positive axillary lymph nodes |  | 0.019 |  | 0.018 |  | 0.057 |  |  |
| ＜2 | 1(reference) |  | 1(reference) |  | 1(reference) |  |  |  |
| ≥2 | 1.993(1.122-3.543) |  | 2.006(1.130-3.561) |  | 1.730(0.984-3.040) |  |  |  |
| Molecular subtype |  | 0.193 |  |  |  | 0.400 |  |  |
| Luminal A | 1(reference) |  |  |  | 1(reference) |  |  |  |
| Luminal B HER2+ | 0.222(0.018-2.684) |  |  |  | 0.296(0.034-2.563) |  |  |  |
| Luminal B HER2- | 0.800(0.102-6.268) |  |  |  | 0.892(0.130-6.121) |  |  |  |
| HER2 enriched | 0.109(0.010-1.178) |  |  |  | 0.318(0.042-2.434) |  |  |  |
| Triple negative | 0.537(0.054-5.312) |  |  |  | 0.162(0.015-1.726) |  |  |  |
| ER status |  | 0.831 |  |  |  | 0.624 |  |  |
| Negative | 1(reference) |  |  |  | 1(reference) |  |  |  |
| Positive | 1.054(0.652-1.702) |  |  |  | 1.313(0.443-3.889) |  |  |  |
| PR status |  | 0.161 |  |  |  | 0.187 |  |  |
| Negative | 1(reference) |  |  |  | 1(reference) |  |  |  |
| Positive | 1.862(0.781-4.442) |  |  |  | 1.829(0.746-4.483) |  |  |  |
| HER2 status |  | 0.091 |  |  |  | 0.185 |  |  |
| Negative (0--++) | 1(reference) |  |  |  | 1(reference) |  |  |  |
| Positive (+++) | 1.883(0.904-3.922) |  |  |  | 1.656(0.786-3.490) |  |  |  |
| Ki-67 status |  | 0.216 |  |  |  | 0.515 |  |  |
| Negative (≤14%) | 1(reference) |  |  |  | 1(reference) |  |  |  |
| Positive (＞14%) | 1.463(0.800-2.675) |  |  |  | 1.243(0.646-2.393) |  |  |  |
| Postoperative chemotherapy |  | 0.024 |  | 0.011 |  | 0.008 |  | 0.007 |
| No | 1(reference) |  | 1(reference) |  | 1(reference) |  | 1(reference) |  |
| Yes | 0.224(0.061-0.820) |  | 0.503(0.296-0.857) |  | 0.122(0.026-0.580) |  | 0.475(0.277-0.814) |  |
| Postoperative radiotherapy |  | 0.948 |  |  |  | 0.385 |  |  |
| No | 1(reference) |  |  |  | 1(reference) |  |  |  |
| Yes | 0.954(0.230-3.951) |  |  |  | 0.580(0.170-1.981) |  |  |  |
| Postoperative endocrine therapy |  | 0.007 |  | 0.001 |  | ＜0.001 |  | 0.001 |
| No | 1(reference) |  | 1(reference) |  | 1(reference) |  | 1(reference) |  |
| Yes | 0.192(0.057-0.643) |  | 0.369(0.206-0.660) |  | 0.034(0.008-0.148) |  | 0.373(0.209-0.666) |  |
| Postoperative targeted therapy |  | ＜0.001 |  | ＜0.001 |  | ＜0.001 |  | ＜0.001 |
| No | 1(reference) |  | 1(reference) |  | 1(reference) |  | 1(reference) |  |
| Yes | 0.104(0.029-0.373) |  | 0.322(0.186-0.556) |  | 0.278(0.148-0.521) |  | 0.305(0.178-0.524) |  |
| Lymph vessel invasion |  | 0.006 |  | 0.001 |  | 0.001 |  | 0.001 |
| No | 1(reference) |  | 1(reference) |  | 1(reference) |  | 1(reference) |  |
| Yes | 5.646(1.653-19.281) |  | 7.768(2.420-24.931) |  | 5.772(1.969-16.920) |  | 2.766(1.525-5.017) |  |
| Neural invasion |  | 0.884 |  |  |  | 0.996 |  |  |
| No | 1(reference) |  |  |  | 1(reference) |  |  |  |
| Yes | 0.911(0.259-3.196) |  |  |  | 0.997(0.263-3.779) |  |  |  |
| p-JNK expression |  | 0.031 |  | 0.003 |  | 0.004 |  | 0.007 |
| Low expression | 1(reference) |  | 1(reference) |  | 1(reference) |  | 1(reference) |  |
| High expression | 0.276(0.086-0.890) |  | 0.214 (0.077-0.597) |  | 0.176(0.053-0.581) |  | 0.194 (0.059-0.633) |  |
